# SLC35B4 Stabilizes c-MYC Protein by O-GlcNAcylation in HCC

**DOI:** 10.3389/fphar.2022.851089

**Published:** 2022-03-02

**Authors:** Tao Jiang, Jinghong Yang, Huohong Yang, Wancheng Chen, Kaiyuan Ji, Yang Xu, Lili Yu

**Affiliations:** ^1^ School of Basic Medical Sciences, Southern Medical University, Guangzhou, China; ^2^ Department of Cardiology, Cardiovascular Key Lab of Zhejiang Province, The Second Affiliated Hospital, Zhejiang University School of Medicine, Zhejiang University, Hangzhou, China; ^3^ Department of Medical Oncology, Key Laboratory of Cancer Prevention and Intervention, Ministry of Education, The Second Affiliated Hospital, Zhejiang University School of Medicine, Hangzhou, China; ^4^ Cancer Center, Zhejiang University, Hangzhou, China

**Keywords:** O-GlcNAcylation, HCC, c-Myc, SLC35B4, nucleotide sugar transporters

## Abstract

UDP-GlcNAc is a sugar substrate necessary for the O-GlcNAcylation of proteins. SLC35B4 is one of the nucleotide sugar transporters that transport UDP-GlcNAc and UDP-xylose into the endoplasmic reticulum and Golgi apparatus for glycosylation. The roles of SLC35B4 in hepatocellular carcinoma (HCC) tumorigenesis remain unknown. We find that the expression levels of SLC35B4 are higher in HCC tissues than adjacent non-tumor tissues. SLC35B4 is important for the proliferation and tumorigenesis of HCC cells. Mechanistically, SLC35B4 is important for the O-GlcNAc modification of c-Myc and thus the stabilization of c-Myc, which is required for HCC tumorigenesis. Therefore, SLC35B4 is a promising therapeutic target for treating HCC.

## Introduction

Liver cancer is one of the most common human cancers and the third leading cause of cancer death in 2020 (Sung et al., 2021). Hepatocellular carcinoma (HCC) is the major type of primary liver cancer (Sung et al., 2021). The main risk factors causing HCC include chronic infection with hepatitis B virus or hepatitis C virus, heavy alcohol use, and non-alcoholic fatty liver disease usually associated with obesity and type 2 diabetes (Rawla et al., 2018). Available therapeutic options for HCC include tumor resection, liver transplantation, percutaneous ethanol injection, and radiofrequency ablation (Karaman et al., 2014; Llovet et al., 2021). However, the effective therapies for advanced HCC are limited due to the lack of understanding of the pathways driving HCC (Jeng et al., 2015). Therefore, to develop more effective HCC therapy, it is important to identify new therapeutic targets that drive HCC tumorigenesis.

O-linked N-acetylglucosaminylation (O-GlcNAcylation) is a common post-translational modification of the serine or threonine residues of protein, which occurs in the endoplasmic reticulum (ER) and Golgi apparatus using UDP-N-acetylglucosamine (UDP-GlcNAc) as the substrate (Hart et al., 2011). This modification is catalyzed by O-GlcNAc transferase (OGT) and removed by O-GlcNAcase (OGA) (Ong et al., 2018). Previous studies have shown that O-GlcNAcylation, the new hallmark of cancer, promotes tumorigenesis *via* multiple mechanisms, including the regulation of cell cycle, chromatin dynamics, and tumor metastasis (Fardini et al., 2013). Elevated O-GlcNAcylation enhances glycolysis by regulating the activity of key glycolytic enzymes, including GLUT1, PFK1, and PGK1 (Bacigalupa et al., 2018; Nie et al., 2020). O-GlcNAcylation has been shown to regulate the stability of c-MYC and HIF-1α *via* direct or indirect manners, which are two oncogenic transcriptional factors critical for tumor progression (Itkonen et al., 2013; Ferrer et al., 2014). Nucleotide sugar transporters (NSTs) are a family of transport proteins that move the glycosylation substrates across the ER or Golgi apparatus membranes (Handford et al., 2006; Hadley et al., 2014). Solute carrier family 35 member B4 (SLC35B4) is one member of NSTs that can transport both UDP-GlcNAc and UDP-xylose (Ashikov et al., 2005). A previous study has demonstrated that SLC35B4 is regulated by oncoprotein YAP1 and promotes gastric cancer development and progression (Liu et al., 2019). However, the roles of SLC35B4 in promoting tumorigenesis remain unknown.

In this study, we showed that the expression levels of SLC35B4 in HCC are higher than normal liver tissues, and the overexpression of SLC35B4 is correlated with the poor prognosis of cancer patients. SLC35B4 knockdown dramatically decreased the proliferation and migration of HCC cells. Mechanistically, while SLC35B4 knockdown did not affect the mRNA levels of c-Myc, it significantly decreased c-Myc protein levels. In addition, we discovered that SLC35B4 knockdown in HCC cells decreased the O-GlcNAcylation of c-MYC that is known to stabilize c-Myc. Therefore, SLC35B4 drives HCC progression by stabilizing c-Myc through O-GlcNAc modification of c-Myc.

## Materials and Methods

### Cell Lines and Cell Culture

Hepatocellular carcinoma cell line (HepG2) was obtained from ATCC. Hepatocellular carcinoma cell line (QGY-7703) was provided by the Pathology Department of Sun Yat-sen University Cancer Center. HEK 293FT was purchased from Thermo Fisher Scientific. All of the cell lines were cultured in Dulbecco’s Modified Eagle Medium (DMEM) containing 10% fetal bovine serum (FBS) and 1% penicillin/streptomycin at 37°C with 5% CO_2_.

### Animals and Human HCC Samples

All animal experiments were performed according to the protocols approved by the Institutional Animal Care and Use Committee (IACUC) of Southern Medical University. For xenograft tumor growth, 5 × 10^6^ cells were injected subcutaneously into the left (control group) and the right (SLC35B4 knockdown group) flanks of the immunodeficient NSG mice (purchased from Shanghai Model Organisms, Shanghai, China. *n* = 6), respectively. For inducible gene knockdown, the drinking water containing 2 mg/L of doxycycline was supplied with doxycycline (20 mg/kg body weight) injected intraperitoneally every day.

After obtaining adequate informed consent, HCC tissue and adjacent normal tissue (ANT, exceeding the edge of the tumor by at least 2 cm) were obtained from HCC patients who underwent curative resection for HCCs in Nanfang Hospital of Southern Medical University, Guangzhou, China, between November 2010 and May 2015. This study was approved by IRB of Nanfang Hospital at Southern Medical University and was performed according to the Declaration of Helsinki (6th revision, 2008).

### Data Analysis From TCGA

GSE25097 dataset analysis was obtained from Gene Expression Omnibus (GEO) database (https://www.ncbi.nlm.nih.gov/geo/query/acc.cgi?acc=GSE25097). Kaplan–Meier survival curves of overall survival in HCC patients were plotted according to the data from a previous study (Nagy et al., 2021).

### Cellular Proliferation and Clonal Formation Assays

HCC cells were digested by 0.25% trypsin at 37°C for 3 min and washed with PBS. For cellular proliferation assay, cells (2,000 cells/well or 5,000 cells/well) were seeded into a 96-well plate. Twenty-four hours later, 10 μl of CCK8 solution was added into each well and incubated at 37°C for 1 h before the absorbance was detected at 450 nm using a microplate reader. Every experiment at least had three repetitions. For clonal formation assay, cells (500 cells/well) were seeded into a 6-well plate and incubated at 37°C for about 2 weeks. After fixation by 100% methanol at room temperature for 15 min, the cell colonies were stained with 0.1% crystal violet for 1 h at room temperature and counted. Every experiment at least had three repetitions.

### Cell Lysis and Western Blotting Analysis

After washing with PBS and harvested, HCC cells were sonicated at 4°C in RIPA buffer (50 mM Tris–HCl pH 8.0, 150 mM NaCl, 1% Triton X-100, 0.5% sodium deoxycholate, 0.1% SDS) containing 1% protease inhibitor cocktail (Thermo Fisher Science). Lysate suspension was obtained after centrifugation at 4°C for 10 min and the protein concentration determined using BCA assay kit (Sigma). Protein denaturation was performed at 100°C for 5 min in 1× sample buffer (Bio-Rad). For Western blotting, the same amount of protein was loaded onto PAGE gel and transferred to a PVDF membrane (Merck) by tank transfer system (Bio-Rad). After being blocked by 5% milk at room temperature for 1 h, primary antibody was incubated with the membrane at 4°C overnight. Immunoblot signal was detected using ChemiDoc Touch Imaging System (Bio-Rad) after the incubation with secondary antibody at room temperature for 1 h.

### Real-Time PCR

Total RNA from cells was extracted using RNeasy Mini Kit (Invitrogen) according to the manufacturer’s instruction. Briefly, cells were lysed in RLT lysis buffer and homogenized using a 1-ml syringe with needle. RNA was purified with RNeasy Mini Kit columns and finally dissolved in RNase-free water. cDNA synthesis from total RNA was performed using PrimeScript RT reagent Kit (Takara) following the manufacturer’s protocol. TB Green Premix Ex Taq II (Takara) was used for Real-Time PCR detection according to the manufacturer’s instruction. Every experiment at least had three repetitions.

### Construct and Lentivirus Production

For SLC35B4 knockdown, two different SLC35B4 shRNA target sequences were synthesized and inserted into pLKO.1-puro vector (Addgene 8453) and tet-pLKO-puro vector (Addgene 21915). For lentivirus production, package plasmid psPAX2 (Addgene 12260), envelop plasmid pMD2.G (Addgene 12259), and the pLKO.1 vectors were co-transfected into HEK 293FT cells. Forty-eight hours after transfection, the supernatant was harvested and concentrated with Lenti-X concentrator (Clontech). The lentivirus was stored at −80°C.

### Cell Migration Assay

For cell migration assay, culture media (500 μl) containing 10% FBS was added into the wells of a 24-well plate with 1 × 10^5^ cells cultured in the inside compartment of a Transwell insert supplemented with DMEM media without FBS. Twenty hours after incubation, the cells attached to the membrane of the Transwell insert were fixed and stained by 0.1% crystal violet. Every experiment at least had three repetitions.

### Immunoprecipitation Assay

HCC cells were harvested and were lysed using Pierce IP lysis buffer (25 mM Tris–HCl pH 7.4, 150 mM NaCl, 1 mM EDTA, 1% NP-40, and 5% glycerol). Protein concentration was determined by BCA assay. For immunoprecipitation assay, 1 mg of total lysate was incubated with anti-c-Myc or anti-O-GlcNAc antibody 4°C overnight. After the incubation with Protein A and G magnetic beads at room temperature for 1 h, the beads were collected and washed 3 to 5 times using lysis buffer. The beads were heated at 100°C for 5 min with 2× sample buffer and stored at −80°C.

### Statistical Analysis

The statistical significance of Kaplan–Meier survival plot was determined by log-rank analysis. The other statistical significance was detected by *t*-test. All of the statistical analyses were performed in GraphPad Prism. *p < 0.05, **p < 0.01, ***p < 0.001, and ****p < 0.0001; n.s. means non-significant.

## Results

### SLC35B4 is Overexpressed in HCC and Correlated With the Poor Prognosis of HCC Patients

**FIGURE 1:**
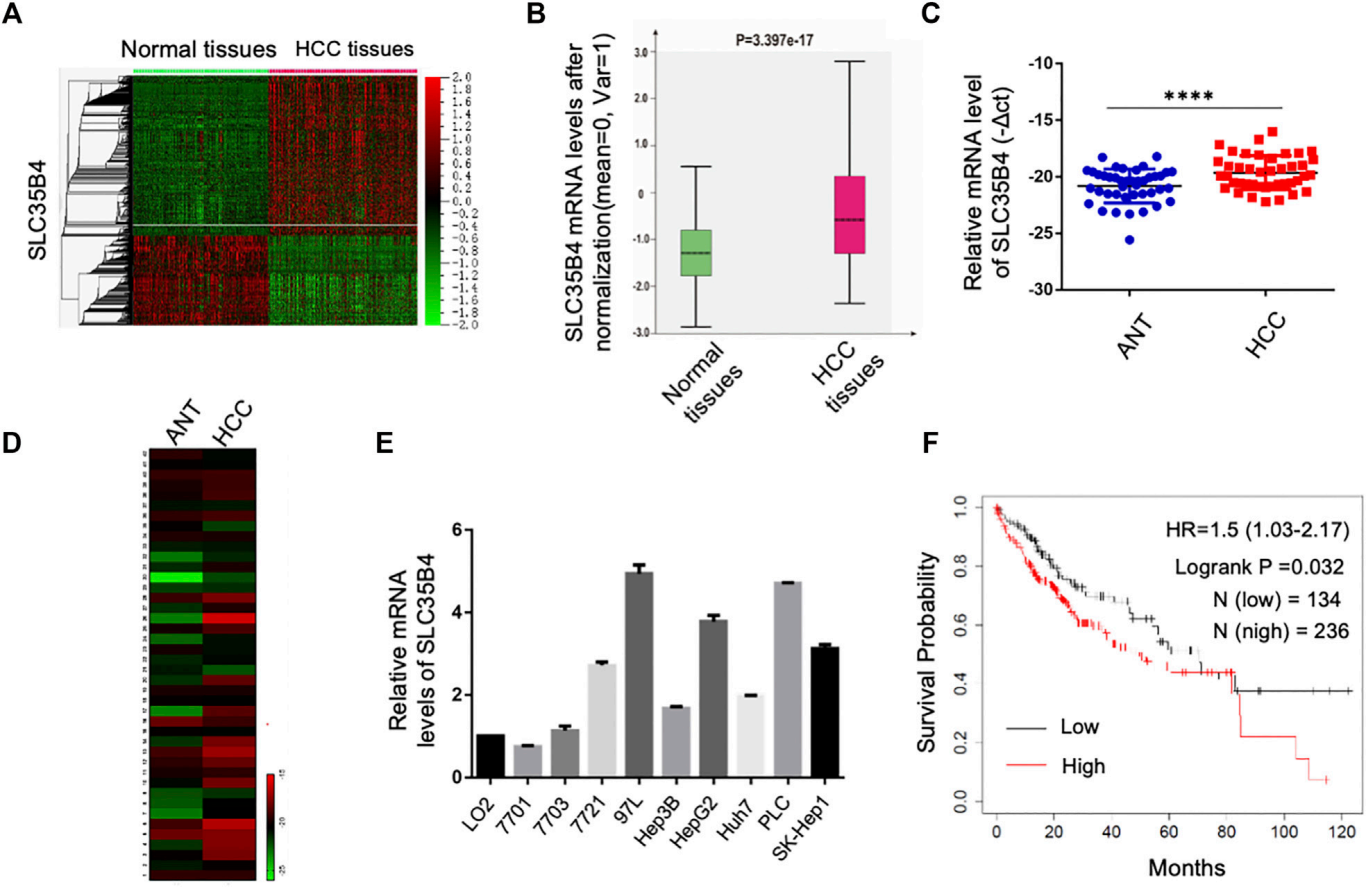
*SLC35B4* is overexpressed in HCC and inversely correlated with prognosis of HCC patients. **(A)** The heat map of mRNA expression profile in 268 non-tumor adjacent tissues and 243 HCC tissues in GSE25097 dataset. The mRNA expression level of *SLC35B4* was indicated by a white line. **(B)** The box plot of *SLC35B4* expression levels in non-tumor tissues (268) and HCC tissues (243) in GSE25097 dataset. *P-*value is indicated. **(C,D)** The relative mRNA expression levels of *SLC35B4* in 42 paired adjacent normal tissues (ANT) and HCC tissues. *P-*value is indicated. **(E)** The mRNA expression levels of *SLC35B4* in HCC cell lines. *N* = 3. Data are presented as mean ± SD. **(F)** Kaplan–Meier survival curve of the overall survival of HCC patients with high and low *SLC35B4* expression levels. The *SLC35B4* expression levels were inversely correlated with the overall survival of HCC patients. *P-*value is indicated.

To examine the potential involvement of SLC35B4 in HCC, the GSE25097 dataset of HCC patients was analyzed, indicating that the SLC35B4 gene was significantly higher in HCC than in adjacent normal tissues (Figures 1A,B). In addition, the analysis of SLC35B4 mRNA levels in 42 paired HCC tissues and adjacent non-tumor tissues further confirmed this conclusion (Figures 1C,D). *SLC35B4* was also widely expressed in HCC cell lines, including HepG2, PLC, and 7703 cells (Figure 1E). The overexpression of *SLC35B4* is correlated with the poor prognosis of the HCC patients (Figure 1F). These findings suggest that SLC35B4 plays important roles in driving HCC development.

### SLC35B4 Promotes the Proliferation and Migration of HCC Cells

**FIGURE 2:**
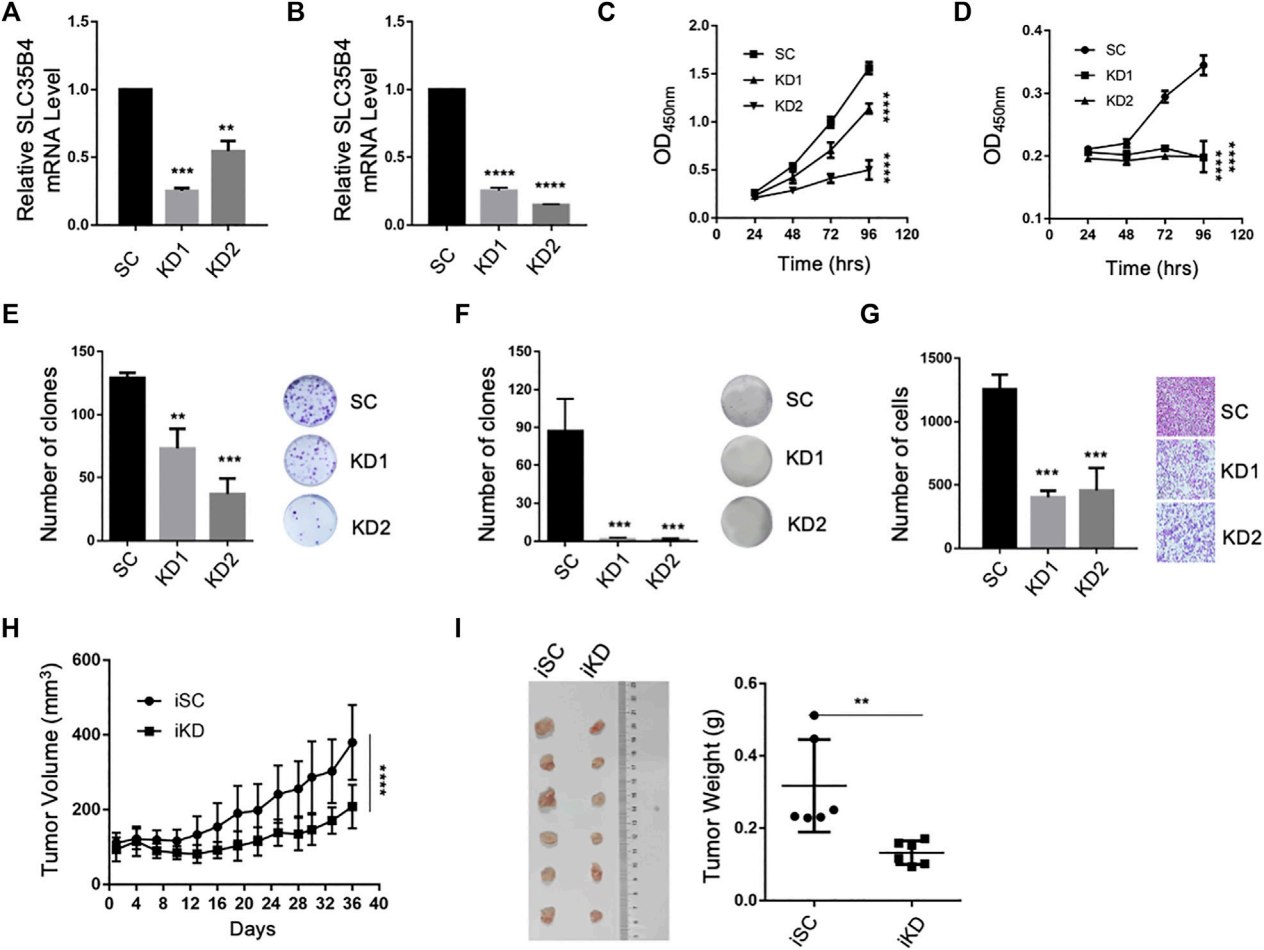
SLC35B4 promotes HCC cell proliferation and migration. **(A,B)** Relative mRNA expression levels of SLC35B4 in HCC cells after *SLC35B4* knockdown. *N* = 3. Data are presented as mean ± SD. **(C,D)** SLC35B4 knockdown significantly decreased the cellular proliferation of HCC cells as determined by CCK8. *N* = 3. Data are presented as mean ± SD. **(E,F)** SLC35B4 knockdown dramatically decreased the clonal formation ability of 7703 **(E)** and HepG2 **(F)** cells. *N* = 3. Data are presented as mean ± SD. **(G)** SLC35B4 knockdown inhibited the migration of HCC cells *in vitro. N* = 3. Data are presented as mean ± SD. **(H)** The volume of xenograft tumors after the inducible knockdown of SLC35B4. iSC, inducible scrambled control; iKD, inducible knockdown. **(I)** The image and weight of xenograft tumors formed by HCC cells with or without inducible SLC35B4 knockdown in NSG mice 37 days after inducible knockdown. *N* = 6. Data are presented as mean ± SD. *p < 0.05, **p < 0.01, ***p < 0.001, ****p < 0.0001.

To study the roles of SLC35B4 in HCC progression, we knocked down SLC35B4 in HCC cell lines 7703 and HepG2 using two specific short hairpin RNAs (Figures 2A,B). The results showed that SLC35B4 knockdown significantly suppressed the proliferation and colony formation capability of HCC cells (Figures 2C–F). In addition, SLC35B4 knockdown suppressed the migration of HCC cells (Figure 2G). Consistent with these findings, the inducible knockdown of SLC35B4 dramatically suppressed tumor growth in the immunodeficient NODSCID mice (Figures 2H,I). These results demonstrate that SLC35B4 is important for HCC tumorigenesis *in vitro* and *in vivo*.

### SLC35B4 Stabilizes c-Myc *via* O-GlcNAc Modification

**FIGURE 3:**
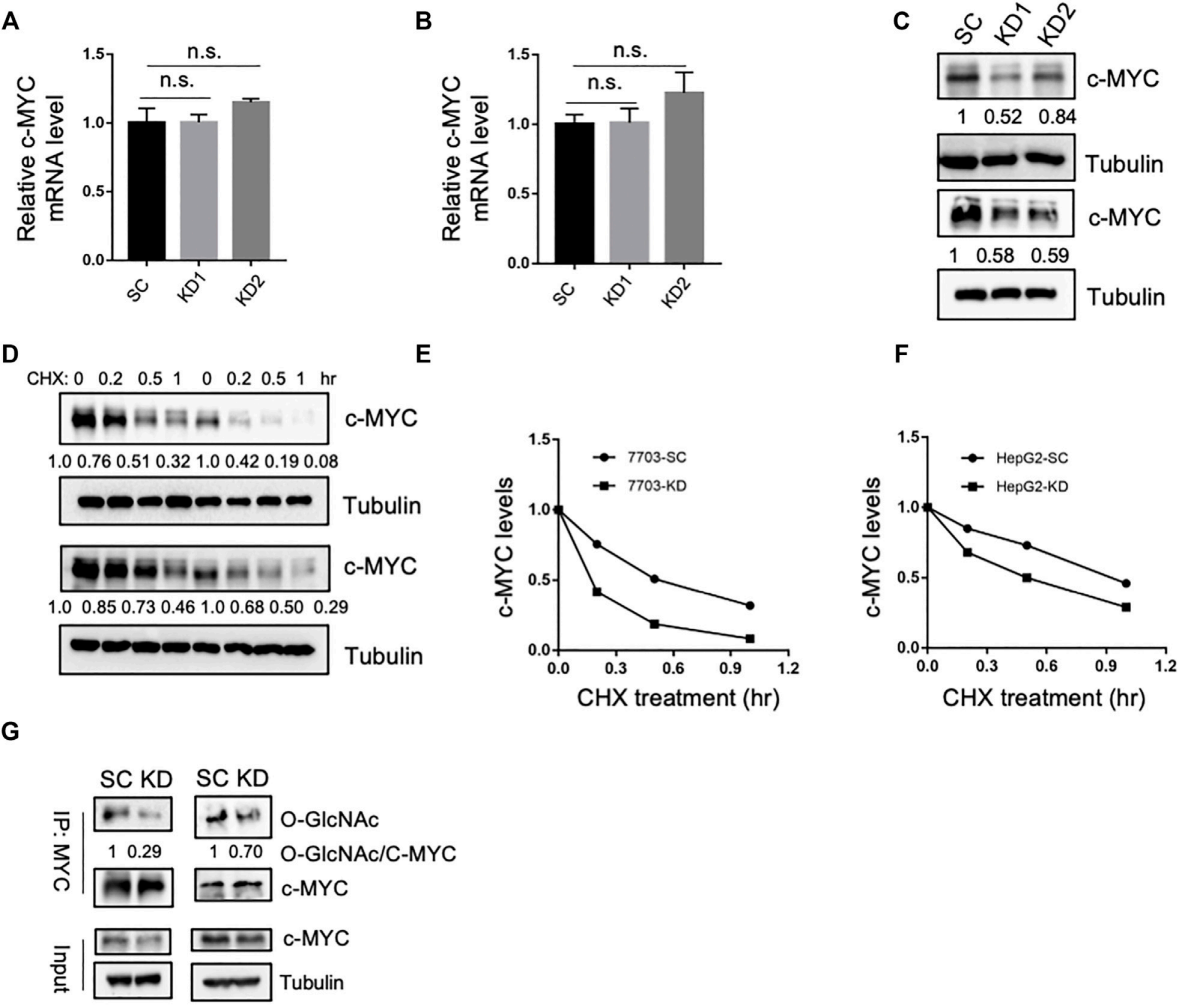
SLC35B4 stabilizes c-Myc *via* O-GlcNAc modification. **(A,B)** Relative mRNA expression levels of c-Myc in 7703 **(A)** and HepG2 **(B)** cells with SLC35B4 knockdown. *N* = 3. Data are presented as mean ± SD. n.s., non-significant. **(C)** The protein levels of c-Myc in 7703 (upper) and HepG2 (bottom) cells with SLC35B4 knockdown. The relative protein levels are indicated. **(D)** The protein levels of c-Myc protein in 7703 (upper) and HepG2 (bottom) cells after CHX treatment. **(E,F)** Quantification of the protein levels of c-Myc in 7703 **(E)** and HepG2 **(F)** cells with or without SLC35B4 knockdown at different time points after CHX treatment. The relative protein levels are indicated. **(G)** The O-GlcNAcylation of c-Myc after SLC35B4 knockdown. c-Myc in 7703 (left) and HepG2 (right) cells with or without SLC35B4 knockdown was immunoprecipitated and the levels of the O-GlcNAcylation were detected with anti-O-GlcNAc antibody. The relative levels of O-GlcNAcylated versus total c-Myc are indicated.

c-Myc is a critical oncogenic transcription factor that directly binds to the promoters of oncogenes and plays key roles in driving cancer progression (Dang, 2012). O-GlcNAc modification of c-Myc protein by O-linked β-N-acetylglucosamine transferase (OGT) can stabilize c-Myc protein in cancer cells (Itkonen et al., 2013). Considering the involvement of SLC35B4 in glycosylation, we speculated that SLC35B4 could regulate the expression of c-Myc. Therefore, we examined the mRNA and protein levels of c-Myc in HCC cells before and after SLC35B4 knockdown. While SLC35B4 KD had no impact on the mRNA levels of c-Myc, it significantly reduced c-Myc protein levels by destabilizing c-Myc (Figures 3A–C). Mechanistically, we showed that SLC35B4 knockdown dramatically decreased the protein stability and the O-GlcNAc modification of c-Myc (Figures 3D–G). Considering the important roles of c-Myc in driving the turnorigenesis of NPC, these results demonstrate that SLC35B4 drives HCC progression by stabilizing c-Myc through O-GlcNAcylation.

## Discussion

HCC remains one of the most lethal malignancies that lack effective therapy. To develop effective therapies and new therapeutics to treat HCC, extensive international effort has been devoted to identify new pathways that drive HCC tumorigenesis. In this study, we provide compelling evidence that SLC35B4, one member of the nucleotide sugar transporters required to transport nucleotide sugars into the ER or Golgi apparatus for protein glycosylation (Bazan et al., 2018), is overexpressed in HCC and drives HCC tumorigenesis. In this context, the knockdown of SLC35B4 inhibits the proliferation and migration of HCC cells *in vitro*, and the acute depletion of SLC35B4 in HCC tumors significantly suppresses the tumor growth *in vivo*. Therefore, SLC35B4 represents a promising new therapeutic target to treat HCC.

To understand the mechanism underlying SLC35B4-dependent tumorigenesis, based on the physiological functions of SLC35B4 in transporting substrates of O-GlcNAc modification into the Golgi and ER, we investigated oncogenic proteins that are regulated by O-GlcNAc modification. Our study demonstrates that SLC35B4 is important to stabilize c-Myc by promoting its O-GlcNAc modification.

c-Myc is a critical oncoprotein that is overexpressed in many types of human cancer and plays key roles in driving tumorigenesis (Pelengaris et al., 2002). Therefore, c-Myc could be an ideal therapeutic target to treat various types of cancers. However, due to the critical roles of c-Myc in normal cellular processes, the complete inactivation of c-Myc will have lethal effects on normal cells (Carabet et al., 2018). Therefore, c-Myc remains an undruggable target in cancer drug discovery. The destabilization of c-Myc protein in human cancers has become a promising strategy to target c-Myc. Therefore, while it remains to be confirmed, our findings that SLC35B4 depletion could destabilize c-Myc provide an alternative approach to target c-Myc in many types of human cancer.

## Data Availability

The original contributions presented in the study are included in the article/Supplementary Material, further inquiries can be directed to the corresponding authors.
